# Clinical and genetic spectrums of 413 North African families with inherited retinal dystrophies and optic neuropathies

**DOI:** 10.1186/s13023-022-02340-7

**Published:** 2022-05-12

**Authors:** Aymane Bouzidi, Hicham Charoute, Majida Charif, Ghita Amalou, Mostafa Kandil, Abdelhamid Barakat, Guy Lenaers

**Affiliations:** 1 Equipe MitoLab, Unité MitoVasc, INSERM U1083, CHU d’Angers, CNRS 6015, Université d’Angers, 49933 Angers, France; 2 Genomics and Human Genetics Laboratory, Institut Pasteur du Maroc, Casablanca, Morocco; 3 Team of Anthropogenetics and Biotechnologies, Faculty of Sciences, Chouaïb Doukkali University, Eljadida, Morocco; 4 Research Unit of Epidemiology, Biostatistics and Bioinformatics, Institut Pasteur du Maroc, Casablanca, Morocco; 5 Genetics, and Immuno-Cell Therapy Team, Mohamed First University, Oujda, Morocco; 6 Service de Neurologie, CHU d’Angers, Angers, France

**Keywords:** Inherited retinal dystrophies, Inherited optic neuropathies, Molecular diagnosis, North Africa, Consanguinity, Phenotypic spectrum, Genetic spectrum

## Abstract

**Background:**

Inherited retinal dystrophies (IRD) and optic neuropathies (ION) are the two major causes world-wide of early visual impairment, frequently leading to legal blindness. These two groups of pathologies are highly heterogeneous and require combined clinical and molecular diagnoses to be securely identified. Exact epidemiological studies are lacking in North Africa, and genetic studies of IRD and ION individuals are often limited to case reports or to some families that migrated to the rest of the world. In order to improve the knowledge of their clinical and genetic spectrums in North Africa, we reviewed published data, to illustrate the most prevalent pathologies, genes and mutations encountered in this geographical region, extending from Morocco to Egypt, comprising 200 million inhabitants.

**Main body:**

We compiled data from 413 families with IRD or ION together with their available molecular diagnosis. The proportion of IRD represents 82.8% of index cases, while ION accounted for 17.8%. Non-syndromic IRD were more frequent than syndromic ones, with photoreceptor alterations being the main cause of non-syndromic IRD, represented by retinitis pigmentosa, Leber congenital amaurosis, and cone-rod dystrophies, while ciliopathies constitute the major part of syndromic-IRD, in which the Usher and Bardet Biedl syndromes occupy 41.2% and 31.1%, respectively. We identified 71 ION families, 84.5% with a syndromic presentation, while surprisingly, non-syndromic ION are scarcely reported, with only 11 families with autosomal recessive optic atrophies related to *OPA7* and *OPA10* variants, or with the mitochondrial related Leber ION. Overall, consanguinity is a major cause of these diseases within North African countries, as 76.1% of IRD and 78.8% of ION investigated families were consanguineous, explaining the high rate of autosomal recessive inheritance pattern compared to the dominant one. In addition, we identified many founder mutations in small endogamous communities.

**Short conclusion:**

As both IRD and ION diseases constitute a real public health burden, their under-diagnosis in North Africa due to the absence of physicians trained to the identification of inherited ophthalmologic presentations, together with the scarcity of tools for the molecular diagnosis represent major political, economic and health challenges for the future, to first establish accurate clinical diagnoses and then treat patients with the emergent therapies.

**Supplementary Information:**

The online version contains supplementary material available at 10.1186/s13023-022-02340-7.

## Background

North Africa encompasses five countries, Morocco, Algeria, Tunisia, Libya and Egypt, which historically and geographically were at the crossroad between Africa, Europe and Asia. This area is delimited by the Mediterranean Sea in the north, and separated from other African countries by the Sahara in the south. The North African population, today numbering 202 million people [1], is mainly occupied by two ethnic groups which are Arab Muslims and Berbers, and a minority of Jews and Christians.

Historically, since the earliest times, the Berber people, also called Amazigh have populated North Africa, before this area became a targeted land of several colonization and demographic migratory movements, first by Phoenicians, then by Romans, Vandals, Byzantines, Arabs, Ottomans, and finally by Europeans during the eighteenth and nineteenth centuries [2].

The interactions between all these populations have made the genetic pool of the North African population very complex and heterogeneous, even among populations living in close geographical areas, or within the same ethnic groups, which have been described by several molecular studies involving mitochondrial DNA, Y chromosome and autosomal markers [3–9]. Jews have appeared in North Africa since the Phoenician influence. Their communities have grown as a result of conversion and admixture with the local Berber population at that time, in addition to several migrations from the middle East and Europe, mainly by the Jews of the Iberian peninsula, after their expulsion from Spain and Portugal between 1492–1497 [10], and were named Sephardic. In 1950s, Jews of North Africa have migrated to the current Israel [10]. Genome-wide studies defined North African Jews as a distinct group, with a strong link to the European and Middle Eastern Jews, and with European non-Jews, rather than North African non-Jews. In addition, they show a high degree of identity of descent segment haplotypes, witnessing the high level of endogamy within the Jewish diaspora [11] that led to high frequency of founder mutations [12].

North African countries show a high rate of endogamy and consanguinity, ranging from 20 to 40% [13, 14], and up to some 60% in some remote villages [15]. This marriage practice is also conserved within North African immigrants who live in other parts of the world, mainly in Europe [16]. Consanguinity increases the chance of bi-allelic associations of a single mutant allele in an individual, and as a consequence, a predominance of diseases related to autosomal recessive transmission. In this respect, an epidemiological study of genetic diseases in the North African population identified 532 pathologies, of which 60% were inherited in an autosomal recessive manner [17].

Vision is the most important of the five senses, and visual impairment has major impacts on the psychological, educational and socioeconomic conditions of affected individuals. Worldwide, 19 million children are estimated to be visually impaired [18]. In developed countries, retinal and optic nerve problems are the major causes of visual impairments, [19–21], up to 24% and 23% respectively, in the UK [21]. Exact epidemiological studies on these disorders are lacking in North Africa, as other vision problems, like refractive errors and cataract are the most common causes of visual impairment in these countries, as in many other regions of the world [22]. Inherited disorders affecting the retina and the optic nerve encompass two heterogeneous groups of diseases that lead to visual impairment, and in some cases, to legal blindness. They are caused by many genes, with autosomal, X-linked and mitochondrial patterns of inheritance. Large cohort studies of inherited retinal dystrophies (IRD) or optic neuropathies (ION) with the spectrum of causal genes and mutations are absent in North African individuals, except for two studies performed on Tunisian patients with IRD [23, 24], or some studies conducted on specific syndromes [25–28]. The majority of genetically characterized individuals are reported in a single family or in few families, or as a part of cohorts located in other countries, mainly in Europe. Recently, a review on the genetics of non-syndromic rod-cone dystrophies in Arab countries disclosed the spectrum of genes and mutations identified in 26 North African Arab families, highlighting the major contribution of variants in *MERTK, RLBP1, RPE65* and *PDE6B* genes [29]. However, in order to access the phenotypic and genetic spectrum of all non-syndromic and syndromic IRD and ION within North African families, we performed a review of the data published in the literature using PubMed and Scopus databases. Different search terms were used (Additional file 1) in combination with the names of North African countries (Morocco OR Algeria OR Tunisia OR Libya OR Egypt OR North Africa OR Maghreb), or terms that refer to patients’ origin (Moroccan OR Algerian OR Tunisian OR Libyan OR Egyptian OR North African OR Maghrebian). Abstracts, full-length and additional materials of articles in English and French published between 1994 and December 2021 and their references’ listing, where both clinical and genetic findings are reported, were examined. Genes and mutations found in families from North Africa were incorporated and are discussed in this review.

### Inherited retinal dystrophies (IRD)

IRD describe a large heterogeneous group of disorders characterized by the dysfunction of the neural retina or retinal pigment epithelium cells [30]. There are at least 280 genes involved in one or more IRD disorders [31]. Indeed, variants in the same gene may lead to different clinical outcomes between and within families. These highly heterogeneous genetic findings gave rise to different clinical manifestations that can be classified on their mode of inheritance, the course of the disease (progressive or stationary), the predominant phenotype (rod-dominant, cone-dominant or macular) and the occurrence of additional systemic symptoms (syndromic IRD) or exclusively eye related symptoms (non-syndromic IRD). More than 80 syndromic forms are described [32], among which, the predominant Usher syndrome, accounting for 20 to 40% of the recessive diseases affecting both the visual and hearing capacities [33].

Large cohort studies across the world on patients with non-syndromic IRD (NS-IRD) showed that *retinitis pigmentosa* is the most frequent phenotype, followed by Stargardt diseases, Leber congenital amaurosis, and cone-rod dystrophies/cone dystrophies, whereas phenotypes like choroideremia, achromatopsia and Best diseases remained at very low frequencies [30, 34–36]. *ABCA4*, *USH2A*, and *EYS* are the most frequently mutated IRD genes, while their mutation frequencies may vary according to the studied populations, their clinical presentations, the recruited cohorts, and their pattern of inheritance [30, 34–36].

### Non-syndromic IRD in North African individuals

**Fig. 1:**
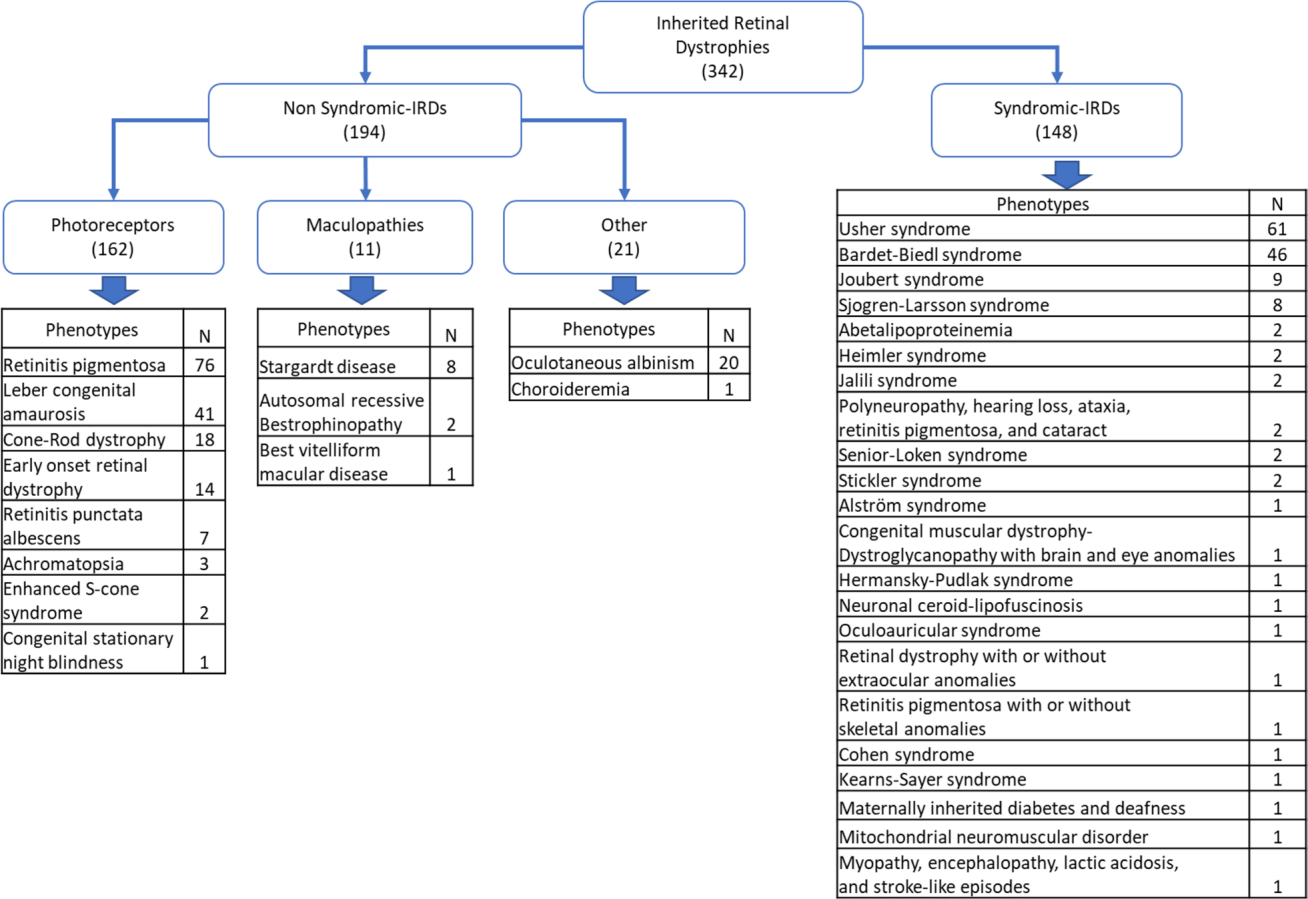
Classification of inherited retinal dystrophies (IRD) and number of affected families (N)

One hundred ninety-four families originating from North Africa with NS-IRD phenotypes have been characterized and reported in the literature, together with the causal genes and mutations. Their phenotypes can be classified into three categories; photoreceptors’ diseases, maculopathies and others (Fig. 1).

**Fig. 2:**
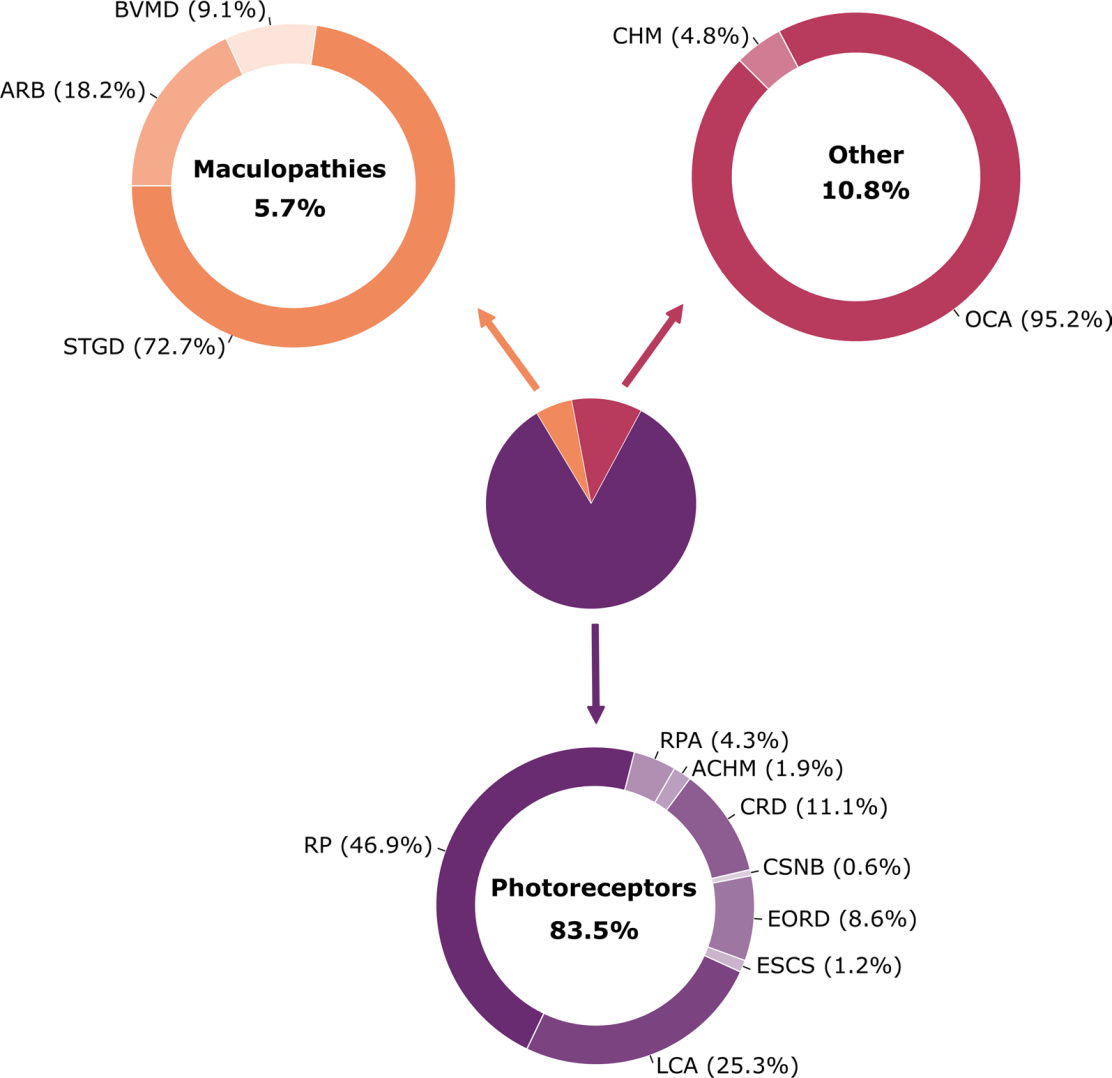
Distribution of the phenotypes of 194 North African families with non-syndromic IRD. According to disease categories in the center chart, and within each category in the outer charts. ACHM: achromatopsia, ARB: autosomal recessive bestrophinopathy, BVMD: Best vitelliform macular dystrophy, CHM: choroideremia, CRD: cone-rod dystrophy, CNSB: congenital stationary night blindness, EORD: early onset retinal dystrophy, ESCD: enhanced S-cone syndrome, LCA: Leber congenital amaurosis, OCA: oculocutaneous albinism, RP: *retinitis pigmentosa*, RPA: *retinitis punctata albescens*, STGD: Stargardt disease

The first group characterized by photoreceptor dysfunctions is the most frequent (83.5%). It includes *retinitis pigmentosa* (46.9%), Leber congenital amaurosis (25.3%), cone and cone-rod dystrophies (11.1%), early onset retinal dystrophies (8.6%), *retinitis punctata albescens* (4.3%), achromatopsia (1.9%), enhanced S-cone syndrome (1.2%) and finally congenital stationary night blindness (0.6%). The second group, consisting in maculopathies, represents 5.7% of the reported families, Stargardt disease being the most frequent (72.7%), autosomal recessive bestrophinopathy and best vitelliform macular dystrophy representing 18.2% and 9.1%, respectively. The third group accounting for 10.8% includes two phenotypes: oculocutaneous albinism which has been described in twenty families and choroideremia in a single family (Fig. 2).

Consanguinity has a major impact among North African families with NS-IRD. 71.4% of the families for whom the relation between parents has been investigated were consanguineous, reflecting the predominance of autosomal recessive (AR) pattern of inheritance. Indeed, 93.3% of these families disclosed mutations on both alleles, while autosomal dominant (AD) transmission has been identified in only three families, with variants in *PRPH2*, *NR2E3* and *PRPF31,* respectively. An X-linked pattern was found in only one family with a *CHM* variant.

**Retinitis pigmentosa (RP, OMIM: 268,000)** is one of the most frequent IRD leading to legal blindness, with a prevalence estimated at 1:4.000 affected individuals worldwide [37]. RP is characterized by a progressive loss of photoreceptors, predominantly the rods associated to the scotopic system, rather than the cones associated to the photopic system. Night blindness or nyctalopia is the earliest symptom in RP patients, followed by a gradual loss of the peripheral visual field leading to a tunnel vision. In addition, pigmentary deposits in the peripheral retina, attenuation of retinal vessels and a waxy pallor of the optic disc represent RP hallmarks [38, 39].

With 76 families, RP is the most common IRD reported in patients originating from North Africa, among which consanguinity rate reaches 63.9%. Almost all RP families (96.1%) present an AR pattern of inheritance, with only three families presenting an AD pattern, and no mutation in an X-linked gene was yet reported.

**Fig. 3:**
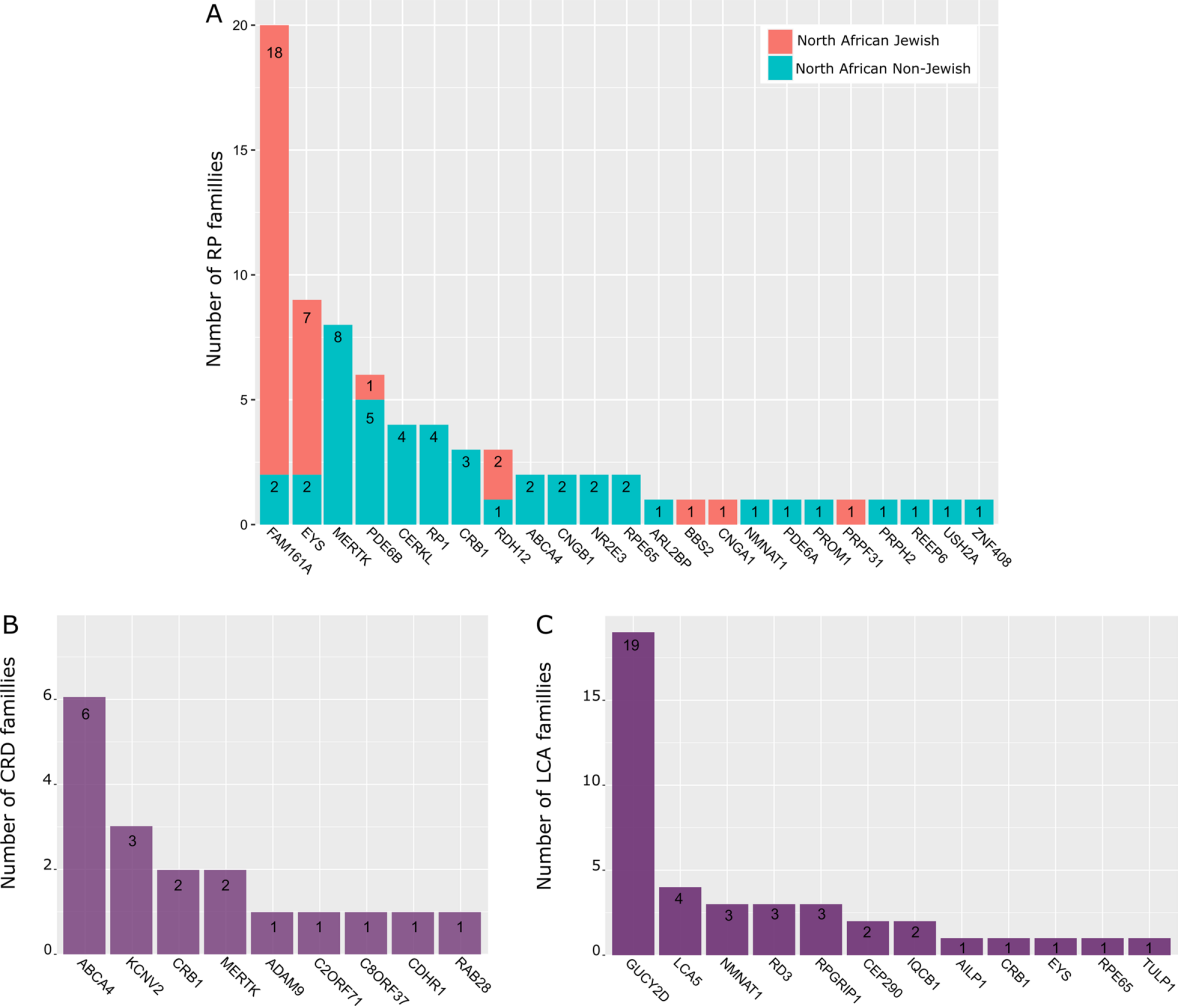
Distribution of the number of families with non-syndromic IRD per causal genes. **A** Families with *retinitis pigmentosa*. **B** Families with cone-rod dystrophy. **C** Families with Leber congenital amaurosis

RP families can be divided in two groups, with different ethnic backgrounds. The first group corresponds to non-Jewish families, characterized by a high genetic heterogeneity, in which variants in twenty different genes were identified in 45 families (Fig. 3a). Among the 20 genes, 5 of them are responsible for 53.3% of the observed phenotypes. *MERTK* is the most frequent gene mutated within 8/45 families, followed by *PDE6B* with 5/45 families and *CERKL* and *RP1* with 4/45 families, respectively. *CRB1* variants have been identified in 3 families, while the other genes were reported only once or twice. A single *MERTK* splice site mutation, c.2189 + 1G > T, is responsible for 4 out of the 8 families reported (Additional file 2), suggesting a founder effect in the North African population [40]. A second frequent mutation c.1133 + 3_1133 + 6delAAGT deletion in *CERKL* seems also to result from a founder effect, as it was reported in 4 unrelated Tunisian families from the same geographical area, sharing a 5.7 cM homozygous region (Additional file 2) [23]. The second group corresponds to a Jewish ethnical background, with 31 reported families originating from North Africa, and only seven genes affected (Fig. 3a), among which *FAM161A* and *EYS* are mutated in 80.6% of cases. Two founder variants in this community were predominant, the *FAM161A* p.Thr452SerfsTer3 found at homozygous state in 14 out of the 18 families, and the *EYS* p.Thr135LeufsTer7 found at homozygous state in 5 of the 7 families (Additional file 2). Both founder variants were not identified in other ethnical groups [41, 42].

Overall, the relative contributions of RP genes in other studies including populations with different ethnical backgrounds [30, 34, 35, 39, 43], disclosed that *USH2A* variants are very often encountered in the world, whereas only one family form Tunisia disclosed heterozygous compound *USH2A* mutations, indicating that *USH2A* variants are not frequently encountered in RP from North Africa.

**Retinitis punctata albescens (RPA, OMIM:136,880):** is a progressive hereditary disease considered to be a subtype of RP, since both presentations share various common ophthalmological symptoms, among them the predominant alteration of rod photoreceptors and night blindness, occurring in early childhood. In addition, absence or rarity of pigmentary deposits in the peripheral retina, moderate narrowing of retinal vessels and frequent macular atrophy are characterizing RPA [44, 45].

A single mutation, corresponding to a 7.36-kb deletion of three exons [7, 8 and 9], within *RLBP1* was found homozygous in 7 families, 6 of them from Morocco and one from Algeria (Additional file 2). Interestingly, a clinical heterogeneity was found in the last family, with a phenotype in between cone-rod dystrophy and RP. This *RLBP1* mutation was found only in individuals from a close geographical origin, and most probably results from a founder effect [45].

**Cone dystrophy (CD)/Cone-Rod dystrophy (CRD) (OMIM:120,970)** describes a heterogeneous group of IRD, characterized by a predominant progressive loss of cone photoreceptors and pigmentary deposits, frequently encountered in the macular area [46]. The prevalence has been estimated between 1:30.000 and 1:40.000 [47]. The earliest symptoms are a decreased of visual acuity and photophobia, a central vision loss marked by a central scotoma in the visual field, and color vision defects occurring in childhood or early adulthood [48]. Additional abnormalities may occur later in most cases, including alterations of rod photoreceptors in CRD and nyctalopia [46].

Eighteen families with a CRD phenotype have been reported with the causal gene in North Africa, 11 with a documented consanguinity (Additional file 2). Fifteen homozygous mutations, and two heterozygous compound mutations in 9 different genes were found. *ABCA4* and *KCNV2* were affected in 9 families, which represent 50% of North African CRD families (Fig. 3b), in accordance with other studies reporting that the majority of autosomal recessive CD/CRD are related to these two genes [48].

**Leber congenital amaurosis (LCA, OMIM:204,000)** is the earliest and most severe retinal dystrophy with a prevalence of 1:80.000, manifesting at birth or during the first 2 years of life [49]. The main clinical LCA features are early blindness or severe visual loss, undetectable ERG responses, nystagmus and amaurotic pupils [50, 51]. LCA patients may have additional clinical features like keratoconus, hypermetropia and cataract [52].

Forty-one families were identified with a LCA phenotype, 36 having a homozygous variant and 5 heterozygous compound variants (Additional file 2). Thirty-one different variants in 12 genes were reported: *GUCY2D* mutations were found in 19 families and represents 46.3% of all cases, followed by *LCA5* in 4 families, *RPGRIP1*, *RD3* and *NMNAT1* in 3 families, *CEP290* and *IQCB1* in 2 families, and *AILP1*, *EYS*, *RPE65, CRB1* and *TULP1,* each in a single family (Fig. 3c).

*GUCY2D* variants on chromosome 17p13.1 were first identified in LCA individuals from 8 North African consanguineous families [53, 54]. Interestingly, studies conducted on a large French LCA cohort disclosed that the majority of LCA families harboring *GUYCY2D* mutations are originating from North Africa [55, 56]. In this respect, the c.389delC, p.Pro130LeufsTer36 mutation identified in North Africa, is responsible for 42.1% of cases with a *GUCY2D* mutation and 19.5% of LCA families. This variant was homozygous in 7 consanguineous families and related to a founder effect (Additional file 2), most probably originating from a Jewish ancestry [56]. Another *GUCY2D* variant, the p.Phe565Ser, was found homozygous in three Algerian families, in addition to a family of Moroccan and Belgian mixed origins (Additional file 2).

**Early onset retinal dystrophy (EORD),** together with LCA represent the most severe form of retinal dystrophies. It is also considered as a moderate and/or delayed form of LCA, constituting a single clinical entity with very subtle differences [57]. Indeed, LCA patients develop symptoms in the first 3 months of life until 1 year of age, with unrecordable or severely diminished ERG responses [58], while EORD patients disclose similar symptoms, but appearing around 5 years of age [59].

Fourteen families were reported with an EORD phenotype, 13 being consanguineous with homozygous variants (Additional file 2). *RPE65* is the most frequently affected gene, representing 50% of EORD families. The predominant c.271C > T (p.Arg91Trp) missense variant was found in 6 families, and is widely described in several studies in individuals from different ethnical backgrounds [60–63]. *RDH12* variants come in the second place with 35.7% of all EORD families with two mutations: the p.Arg65Ter found in 2 Tunisian families from the same region and the p.Leu99Ile found in 3 Jewish North African families. The last one is known for its founder effect, and was already described among Caucasian patients [64, 65].

**Stargardt disease (STGD, OMIM:248,200)** is the most frequent macular dystrophy characterized by central visual loss in childhood, with a prevalence ranging from 1:8.000 to 1:10.000 [66]. Clinical features include beaten-bronze appearance or bull’s eye maculopathy, progressive atrophy of the macula and yellowish flecks in the posterior pole of the retina [67]. Ninety-five % of STGD cases are inherited as a recessive trait and are caused by *ABCA4* variants. The remaining 5% cases are caused by dominant variants in *ELOVL4* and *PROM1*, and are responsible for STGD3 (OMIM:600,110) and STGD4 (OMIM:603,786), respectively [66, 68].

Eight Tunisian recessive families were characterized with STGD phenotype, and consanguinity was reported among 7 of them, with homozygous *ABCA4* variants. 62.5% of cases are explained by two variants, the p.Arg681Ter mutation found in three families, twice in a homozygous state and once in compound heterozygous state, and the p.Glu1087Lys mutation which was found homozygous in 2 families (Additional file 2).

Two additional maculopathies were characterized within 3 consanguineous families with homozygous *BEST1* variants, 2 families with autosomal recessive Bestrophinopathy (OMIM:611,809) and one with Best vitelliform macular dystrophy (BVMD) (OMIM:153,700) (Additional file 2). BVMD is the second cause of maculopathies after STGD [69], and is often inherited in an AD mode, although the AR mode was also reported [70, 71].

Other NS-IRD have been also reported, and are mainly congenital stationary IRD. Achromatopsia (OMIM:216,900), also called rod monochromatism, characterized by dysfunction of the cone photoreceptors was observed in 3 consanguineous families displaying mutations in *CNGA3*, *CNGB3* and *GNAT2,* respectively (Additional file 2). The Enhanced S-cone syndrome (OMIM:268,100), in which biallelic variants in *NR2E3,* and more rarely in *NRL* lead to increased number of S-type cone photoreceptors and degeneration of L/M-type cones and rod photoreceptors. This phenotype disclosed by electroretinography measurements [72], was described in 2 families with homozygous and heterozygous compound *NRL* variants (Additional file 2). Congenital stationary night blindness (OMIM:613,216) and Choroideremia (OMIM:303,100) were also described in 2 consanguineous families, with a homozygous *TRPM* variant and a hemizygote *CHM* variant, respectively (Additional file 2). Oculocutaneous albinism (OMIM:203,100) is also frequent, in particular among Jewish of Moroccan origin. Twenty families were reported, one with *SLC45A2* and 19 with *TYR* variants, among which 9 with the p.Gly47Asp variant in a homozygous state and 4 in a heterozygous state (Additional file 2).

### Syndromic IRD in North African population

**Fig. 4:**
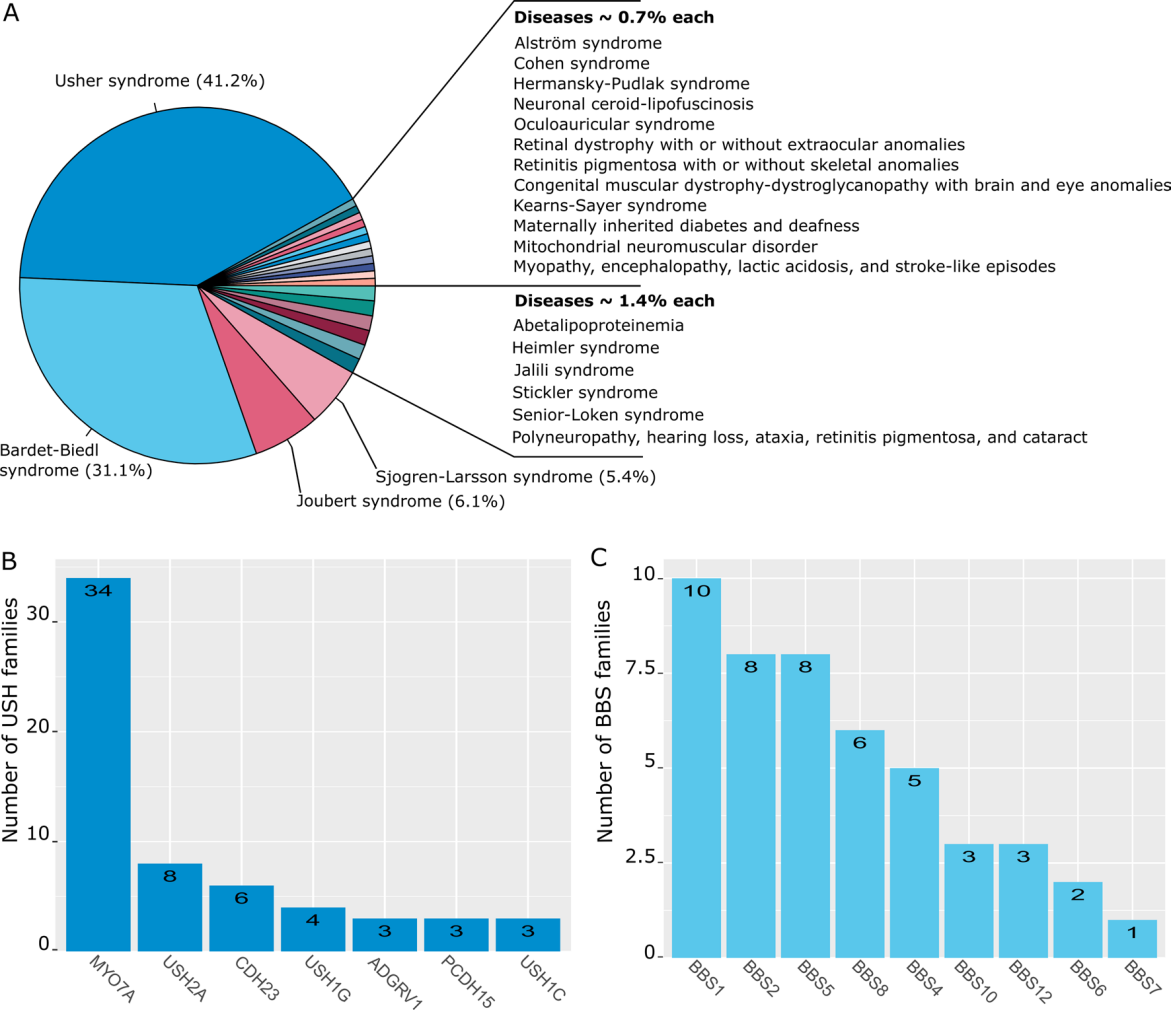
Genetic and phenotypic spectrum of North African families with syndromic IRD. **A** Distribution of the phenotypes of 143 families with syndromic IRD. **B** distribution of families with Usher syndrome phenotype per causal gene. **C** Distribution of families with Bardet-Biedl syndrome phenotype per causal gene

At least, 24 different disorders associated to syndromic inherited retinal dystrophies (S-IRD) were reported and genetically characterized in individuals originating from North Africa. These disorders consist in ciliopathies, in which the Usher and Bardet Biedl syndromes occupy 41.2% and 31.1% of all cases, respectively (Fig. 4a). In addition, diseases related to inborn errors of metabolism, mitochondrial defects related to mitochondrial DNA variants, and associated to cerebellar ataxia were reported.

**Usher syndrome (USH)** is the most common syndromic IRD that associates RP to hearing impairment. It accounts for 10–20% of all RP cases and up to 50% of deaf-blind patients [37, 73]. Three clinical subtypes (USH1-3) are described based on the age of onset, degree of hearing loss (profound, severe or moderate), presence or absence of vestibular dysfunction and age of RP onset. USH1 (OMIM:276,900) is the most severe form, characterized by a profound congenital hearing loss, vestibular dysfunction and prepubescent RP onset. USH2 (OMIM:276,901) consists in a congenital and less severe deafness, without vestibular dysfunction, and a postpubescent RP onset. USH3 (OMIM:276,902), is characterized by a progressive and mild deafness, variable presentation of vestibular dysfunction and variable age of RP onset [74, 75]. Variants in *USH* genes are characterized by high clinical heterogeneity that can cause either USH, non-syndromic RP and non-syndromic deafness. Many studies including North African individuals with non-syndromic deafness have evidenced *USH* variants, but only few associated to RP [76–82].

In North Africa, 61 families have been clinically and genetically characterized with Usher syndrome. This number might be underestimated because individuals have not systematically access to both ophthalmological and audiological examinations to confirm the USH diagnosis. The consanguinity rate within USH families is 77.3%, and 96.7% of them have variants identified on both alleles. Distribution of USH phenotypes is predominated by USH1 (82%). *MYO7A* is the most prevalent gene, with variants identified in 34 families, followed by *USH2A* in 8 families, *CDH23* in 6 families, *USH1G* in 4 families, *ADGRV1*, *PCDH15* and *USH1C* in 3 families each (Fig. 4b).

Five variants were found routinely, four in *MYO7A* and one in *USH2A* genes (Additional file 3), with different occurrences among non-Jewish and Jewish families. Two *MYO7A* splice site mutations are involved in 10 non-Jewish families: one, the c.2283-1G > T mutation was found homozygous in 4 families and heterozygous in 2 families, and is present in Moroccan, Algerian and Tunisian individuals [26, 82–84]. The second *MYO7A* variant, c.470 + 1G > A was found homozygous in 4 families of Algerian and Tunisian origins [26, 81, 85, 86]. Together, these 2 common variants might reflect founder effects in close geographic areas.

Among Jewish families, the p.Ala826Thr *MYO7A* variant was described in 8 families of Moroccan and Algerian origins, while a large *MYO7A* deletion p.Gln2119_Lys2215del was observed in 3 Tunisian families [85, 87, 88]. In addition, the p.Arg334Trp *USH2A* variant was found in several Moroccan and Tunisian Jewish families with a possible founder effect [87, 89, 90].

**Bardet-Biedl syndrome (BBS, OMIM:209,900)**, is a multisystemic disorder characterized by symptoms in several organs that represents 5–6% of syndromic RP cases [37]. The wide heterogeneity of symptoms is divided into 2 categories based on clinical features and frequencies, encountered in patients. Primary features present at high frequencies are RP, polydactyly, obesity, genital and renal anomalies and learning difficulties. Secondary less frequent features are speech and/or development delay, diabetes mellitus, dental anomalies, congenital heart diseases and additional peculiar symptoms [91, 92]. The combination of 3 to 4 primary features associated to 2 secondary features are required to establish a BBS clinical diagnosis [91].

Forty-six families originating from North Africa were reported, among which 28 in the Tunisian population (Additional file 3). Homozygous variants were found in the majority (93,5%) of these families. Thirty-four variants in 9 genes were reported; *BBS1* in 10 families, *BBS2* and *BBS5* in 8 families each, *BBS8* in 6 families, *BBS4* in 5 families, *BBS10* and *BBS12* in 3 families each, while variants in *BBS6* and *BBS7* were only associated twice and once to BBS phenotypes, respectively (Fig. 4c). *BBS1* is the most frequently mutated gene, accounting for 23.2–33.6% of all BBS families, according to different studies performed across the world in multiethnic cohorts [93–95]. In contrast, *BBS2*, *BBS5* and *BBS8* appear more frequently mutated in the North African population than in the rest of the world [94–97].

Two recurrent variants are found frequently in Caucasian BBS families, the p.Met390Arg contributing to 73.3–82.6% of *BBS1* families [93, 95, 98], and the p.Cys91fsX95 contributing to 48.3% to 88.8% of *BBS10* families [95, 96]. The first was found only once in a Tunisian family in a homozygous state, while the second one was never reported in North Africa. Conversely, some *BBS* variants were frequent in Tunisian families with a common haplotype [28], the c.459 + 1G > A in *BBS8* and the p.Arg189Ter in *BBS2*, the latter also encountered in Algeria, witnessing a migration between these 2 countries. In addition, the c.1473 + 4A > G and c.448C > T in *BBS1*, and ex4-5-6del in *BBS4* variants were evidenced in Algerian and Tunisian families, whereas the c.149T > G and c.123delA in *BBS5* variants also frequent, were restricted to Tunisia (Additional file 3).

**Senior-Loken syndrome (SLS, OMIM:266,900)** and **Joubert syndrome (JBTS, OMIM:213,300)** are 2 inherited nephronophthisis related ciliopathies, representing a heterogeneous group of disorders characterized by autosomal recessive cystic kidney and renal anomalies. SLS with a prevalence of 1:1.000.000 is defined by the combination of retinal degeneration ranging from RP to LCA associated to nephronophthisis [99]. JBTS is defined by primary features including hypotonia/ataxia, developmental delay, oculomotor apraxia, mental retardation and breathing anomalies [100], and often associated to retinal degeneration [101–103].

Only two SLS families with mutations in *NPHP4* and *IQCB1* have yet been reported. Conversely, individuals with JBTS phenotype originating from North Africa, especially from Egypt, were well characterized in several studies [104–109], although few of them had a retinal dystrophy. Pathological variants (Additional file 3) responsible for JBTS associated with retinal dystrophies involve *AHI1* in 4 families, followed by *CEP290* and *INPP5E* in 2 families each, and *NPHP1* in one family. *CEP290* is the most frequent JBTS causative gene, but variants in *AHI1* and *INPP5E* are the most frequently associated to retinal dystrophies [102–104, 108]. Finally, only 10% of variants in *NPHP1*, were reported to cause RP [101]. In addition, Alstom and Cohen syndromes, 2 other ciliopathies have been reported, but only in one family each, harboring mutations in *ALMS1* and *VPS13B,* respectively.

**Sjogren-Larsson syndrome (SJLS, OMIM:270,200)** is caused by mutations in *ALDH3A2*, encoding the fatty aldehyde dehydrogenase (FALDH) enzyme, implicated in the metabolism of fatty alcohol [110]. SJLS consists in 3 main symptoms, ichthyosis, mental retardation and spastic diplegia [110], although, additional abnormalities including retinal manifestations are widely described [25, 111–113]. A single study conducted in 25 Egyptian SJLS families has evidenced 8 families with retinal abnormalities consisting in yellowish retinal dots [25]. Three recurrent variants, p.Ser365Leu, p.Arg9ter and p.Gly400Arg contributes for 25%, 12% and 12% of all cases, respectively. Patients harboring the latter 2 variants shared a common haplotype, supporting a founder effect. Interestingly, families harboring the same variant exhibited different retinal manifestations, most probably related to a genetic modifier effect. Additional North African families without retinal defect were further reported [114–118].

**Abetalipoproteinemia (ABL, OMIM:200,100)** is a systemic disease caused by errors in lipid metabolism that manifests by the absence of apolipoproteins b, very low density of lipoproteins and low density lipoproteins in the plasma [119], in addition to diarrhea caused by fat mal absorption, acanthocytosis and retinal dystrophy [112, 119]. Five ABL families of Tunisian origin were reported, 2 of them presenting retinal degeneration and homozygous *MTTP* variants (Additional file 3).

**Heimler syndrome (HS)** is an autosomal recessive peroxisomal biogenesis disorder (PBD) characterized by sensorineural deafness, enamel hypoplasia, nail abnormalities and occasionally retinal pigmentation [120]. PBD represent a group of disorders characterized by anomalies in peroxisome assembly and/or biochemical functions. Peroxisomes are including over 70 enzymes that ensure several metabolic pathways critical for normal cell functioning [121]. HS, the mildest form of the PBD spectrum, is caused by hypomorphic *PEX1* variants defining the Heimler syndrome type 1 (OMIM:234,580) or by *PEX6* variants defining the Heimler syndrome type 2 (OMIM:616,617) [120]. Variants in both genes have been identified in 2 consanguineous families from Moroccan and Egyptian origins, respectively (Additional file 3).

**Familial isolated vitamin E deficiency (AVED, OMIM:277,460)** is an autosomal recessive cerebellar ataxia characterized by low vitamin E level in the serum. Clinical symptoms are resembling those of Friedreich ataxia (FA) with dysarthria, progressive limb and gait ataxia, absent tendon reflexes and skeletal abnormalities [122]. Absence of diabetes and cardiomyopathy are the main characteristics that differentiate AVED from FA [123]. Since the first description by Burk et al. [124], several North African families were described, allowing the identification of the causative locus at 8q and later of variants in the *TTPA* gene, as responsible for the phenotypes [125–128]. A specific *TTPA* frameshift corresponding to an adenine deletion at position 744 (c.744delA) was found in the majority of AVED patients from Mediterranean countries, which correlates with an ancient founder effect [122, 126, 128–134]. Association between AVED and RP has been first described in patients with the p.His101Gln variant causing a less severe and a latter onset presentation [135, 136]. Further studies of North African cohorts revealed that individuals with the c.744delA variant may also present RP, with an incidence varying from 0% in Algeria, 4.4% in Tunisia to 27% in Morocco [122, 131–133]. Other variants have been also reported in AVED North African individuals, but no retinal abnormality was associated [122, 137].

**Spinocerebellar ataxia type 7 (SCA7, OMIM:164,500)** refers to an entity of progressive cerebellar ataxia associated with a cone-rod macular dystrophy, and also known as autosomal dominant cerebellar ataxia type II. SCA7 is caused by a CAG triplet expansion in *ATXN7* at the 3p14.1 locus. The normal allele contains 7 to 17 repeats, and up to 35 repeats in asymptomatic patients, whereas symptomatic patients presents 36 or more CAG triplets [138–141]. *SCA7* has been described in several North African families, with extended CAG repeats [139, 142–144].

Other occasional syndromic IRD were identified in 9 consanguineous families with 7 different syndromes, 2 families with Stickler syndrome (OMIM:614,134) harboring a single *COL9A1* variant, 2 families with Jalili syndrome (OMIM:217,080) with 2 different *CNNM4* variants, one family with congenital muscular dystrophy-dystroglycanopathy with brain and eye anomalies (OMIM:616,538), Hermansky-Pudlak syndrome (OMIM:614,072), oculoauricular syndrome (OMIM:612,109), retinal dystrophy with or without extraocular anomalies (OMIM:617,175), *retinitis pigmentosa* with or without skeletal anomalies (OMIM:250,410), harboring variants in *DAG1*, *HPS3*, *HMX1*, *RCBTB1* and *CWC27,* respectively (Additional file 3).

Finally, retinal alterations like pigmentary retinopathy and maculopathy can be further associated to disorders related to mitochondrial genome mutations. Pigmentary retinopathy is frequently associated to the Kearns-Sayer syndrome (KSS), the neuropathy, ataxia and retinitis pigmentosa (NARP), the mitochondrial encephalomyopathy, lactic acidosis, and stroke like episodes (MELAS) and the maternally inherited deafness and diabetes (MIDD) [145, 146]. Studies performed on North African individuals affected by mitochondrial diseases and retinal manifestations are rare, and only 4 families were reported with KSS, MIDD, MELAS and a mitochondrial neuromuscular disorder, with mtDNA specific mutations or large deletions (Additional file 6).

### Inherited optic neuropathies

Inherited optic neuropathies (ION) define a group of diseases characterized by the dysfunction of the optic nerve as a result of the loss of retinal ganglion cells and their axons [147]. Familial expression of the disease can help its diagnosis, although genetic analysis can also reveal the etiology of the disease, even in absence of familial history. Typically, ION manifest as bilateral symmetric central visual loss with a central scotoma, resulting from the injury of the papillomacular nerve fibers. In this context, its always crucial to rule out IRD that can interfere with primary IONs, especially the CRD affecting the central visual field, in which a pallor of the optic nerve head can also be found [148]. Optic atrophy can be the only manifestation of the IONs in non-syndromic ION (NS-ION), or associated to additional various symptoms, defining syndromic ION (S-ION) [149, 150]. All patterns of inheritance were observed in ION: autosomal dominant or recessive, X-linked and maternally transmitted by the mitochondrial genome. In general NS-ION are less heterogeneous compared to IRD, as only 12 loci have been mapped, 6 being dominant, 5 recessive and 1 X-linked, with 4 genes still to be identified.

Leber hereditary optic neuropathy (LHON;OMIM:535,000) and dominant optic atrophy (DOA;OMIM:165,500) are the 2 most common optic neuropathies worldwide, with a prevalence ranging around 1/15.000 to 1/50.000 for each, depending on the geographic area [147, 149, 151, 152]. LHON was first described by Leber [153], with a maternal mode of inheritance associated to a mitochondrial DNA (mtDNA) variant [154], then to many additional variants of the mtDNA [155]. DOA or Kjer’s optic atrophy was described first in 1959 [156], as an infantile optic atrophy with autosomal dominant mode of inheritance, characterized by a progressive loss of visual acuity, tritanopia, a pallor of the nerve optic heads, mainly in the temporal area, and a centrocoecal scotoma [147, 150, 157]. Genetic studies associated DOA to chromosome 3q28 [158], then to *OPA1,* encoding a mitochondrial dynamin-like GTPase essential for fusion of the mitochondrial network, the production of ATP and the control of apoptosis [157, 159–161]. *OPA1* is mutated in more than 70% of DOA individuals [162], followed by *WFS1* and *ACO2* genes. Recessive loci were also described, as OPA6, OPA7, OPA9, OPA10 and OPA11 [163–167].

Interestingly, all DOA genes discovered so far, encode proteins involved in mitochondrial physiology, with functions involved in their biogenesis, network structure, calcium homeostasis and respiration [168–171], thus emphasizing the frailty of the optic nerve to mitochondrial dysfunctions.

**Fig. 5:**
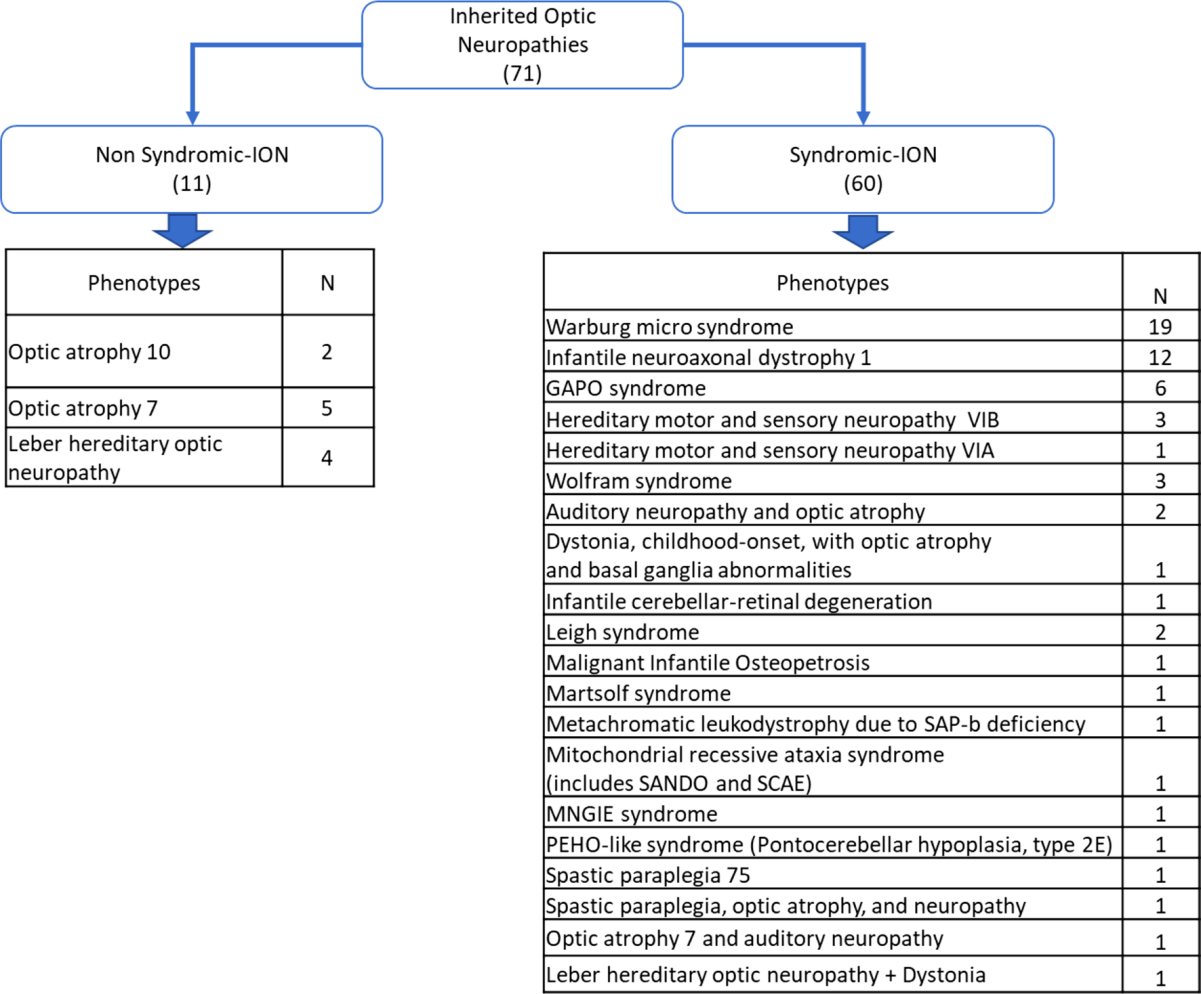
Classification of inherited optic neuropathies (ION) and number of affected families (N)

**Fig. 6:**
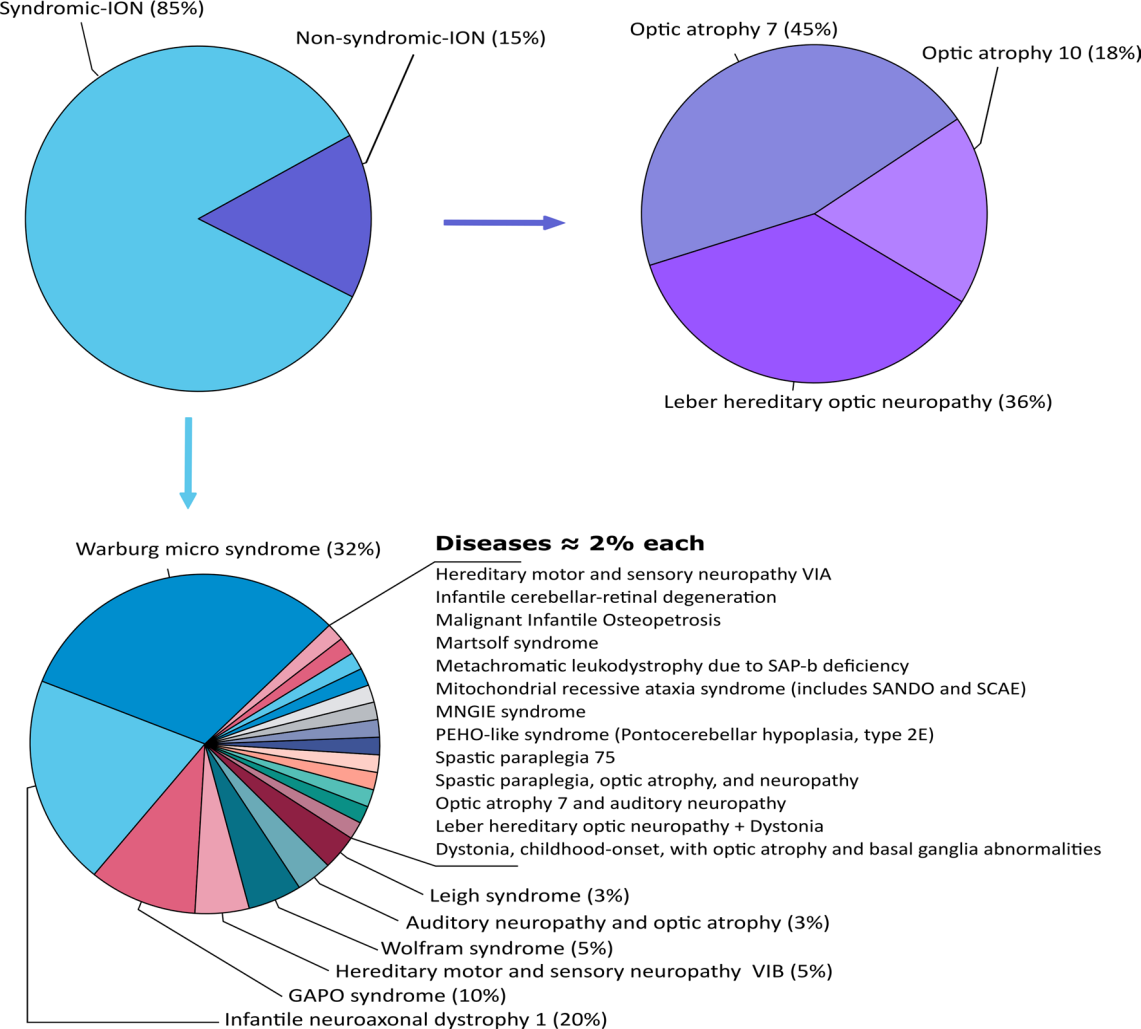
Distribution of the phenotypes of the 71 North African families with ION. According to the disease presentation (syndromic or non-syndromic) in the center chart, and within each category in the outer charts

In the following section, we present the phenotypic and the genotypic spectrums associated with ION in 71 North African families (Figs. 5 and 6).

### Non syndromic inherited optic neuropathies in North African families

**Leber hereditary optic neuropathy (LHON, OMIM:535,000):** is the first disease which was associated to a mtDNA mutation, and now the most frequent among mitochondrial diseases [154, 172]. LHON is a non-syndromic optic neuropathy affecting predominantly men, characterized by a subacute, rapid and painless central visual loss in one eye, then of the fellow eye within weeks or months in the vast majority of patients. Ophthalmological examination discloses a centrocaecal scotoma in the visual field, temporal pallor of the optic nerve head and frequent dyschromatopsia [172–174]. 90–95% of patients with LHON phenotype harbor one of the 3 common mutations m.11778G > A, m.14484T > C, and m.3460G > A affecting the *ND4*, *ND6* and *ND1* genes, respectively [154, 174–176]. Only, the first 2 mutations were identified in individuals from 4 families originating from North Africa, 3 with the m.11778G > A and one with the m.14484T > C variant [177–179] (Additional file 6).

Other clinical symptoms, such as ataxia, dystonia, and encephalopathy can be associated with LHON, namely LHON *plus* phenotype [180], which was reported associated to the m.G14459A variant found in an Algerian family presenting LHON associated to dystonia [181, 182].

Other reported NS-ION cases are related to nuclear DNA variants (Additional file 4). Although the majority of ION individuals reported worldwide present an AD pattern of inheritance, with *OPA1* as the most frequently mutated gene, no ION with a dominant transmission has yet been reported in North Africa. Indeed, 8 consanguineous families with an AR pattern of inheritance were reported with variants in *TMEM126A* and *RTN4IP1* genes. *TMEM126A* was the first gene identified in a large consanguineous Algerian family, harboring a homozygous nonsense mutation p.Arg55Ter. The same mutation was found in two additional Moroccan and one Tunisian families [164]. Genotype analyses revealed a common haplotype within these families, witnessing a founder effect that most probably appeared 80 generations ago. The same *TMEM126A* nonsense variant was further identified in an Algerian consanguineous family with optic atrophy and auditory neuropathy [183] and a Moroccan consanguineous family with isolated optic atrophy [184]. Two additional families presented *RTN4IP1* homozygous variants: the p.Arg103His and the p.Ile362Phe, respectively [163, 185]. Although *RTN4IP1* variants can cause very severe syndromic diseases, North African families presented only a NS-ION phenotype.

### Syndromic inherited optic neuropathies in North African families

**Wolfram syndrome (WFS, OMIM:222,300)** describes a syndromic neurodegenerative disease characterized by optic atrophy, diabetes mellitus, diabetes insipidus and deafness, also called (DIDMOAD) [186]. Additional neurological, endocrinological and other peculiar symptoms can be associated [186, 187]. Variants in 2 nuclear genes *WFS1* and *CISD2*, in addition to heteroplasmic deletions of mtDNA were reported to cause this phenotype [188–191]. Three North African families were reported so far with a WFS phenotype, 2 of them are consanguineous and carry a homozygous mutation in *WFS1* and *CISD2* (Additional file 5). The third family, which presents WFS associated to a cardiomyopathy, was reported with multiple mtDNA deletions in addition to the homoplasmic m.3337G > A mitochondrial variant (Additional file 6). Additional WFS families were also described, but without molecular diagnosis [192–194].

**Leigh syndrome (LS, OMIM:256,000):** is a syndromic neurodegenerative disease characterized by bilateral necrotic lesions in brainstem, basal ganglia, thalamus, cerebellum and spinal cord [195]. LS is a very heterogeneous disease, with more than 35 nuclear and mitochondrial genes involved, thus inherited as AR and maternal mitochondrial or X-linked patterns [196]. Most LS genes are implicated in mitochondrial energy production, encoding proteins involved in the formation of the respiratory chain complexes or their assembly. Ophthalmological abnormalities are frequently described in LS (79% to 82%), including refraction errors, ptosis, strabismus, nystagmus, optic atrophy and pigmentary retinopathy. However the last two abnormalities occur only in 17 to 22.5% and 15 to 22.5% of all individuals, respectively [197, 198]. Several studies described North African LS individuals [195, 199–202], but only 2 families had optic atrophy with either a *SURF1* variant c.516_517delAG (Additional file 5), or a heteroplasmic variant m.9478T > C in the mitochondrial genome (Additional file 6).

**Hereditary motor and sensory neuropathy type VI (OMIM:601,152)** is a neurological disorder characterized by distal neuropathy and optic neuropathy [203]. Mutations in 2 genes, *MFN2* and *SLC25A46*, were reported with an AD and AR inheritance pattern. They encode outer mitochondrial membrane proteins acting on mitochondrial dynamics [204, 205]. Four North African families were reported so far, 3 consanguineous families with *SLC25A46* homozygous variants and one with a dominant *MFN2* variant (Additional file 5).

Five additional families with 4 different phenotypes were reported with variants in genes encoding proteins that ensure mitochondrial physiology (Additional file 5). They affect *FDXR*, which encodes a ferredoxin reductase implicated in Fe-S cluster formation, [206, 207], *MECR*, which encodes a mitochondrial trans-2-enoyl-Coa reductase enzyme essential for fatty acid synthesis and respiratory competence [208], *POLG*, which encodes the mitochondrial polymerase gamma ensuring mtDNA replication and repair [209], and *ACO2*, encoding the mitochondrial aconitase converting citrate to isocitrate in the Krebs cycle [166].

**GAPO syndrome (OMIM:230,740)** is a AR disorder characterized by growth retardation, alopecia, pseudo-anodontia and frequent optic atrophy, caused by *ANTXR1* variants [210]. Six consanguineous Egyptian GAPO families were reported in addition to one family without optic atrophy [211], all presenting homozygous *ANTXR1* variants (Additional file 5).

**Infantile neuroaxonal dystrophy (INAD, OMIM:256,600)** is an early onset AR neurodegenerative disorder appearing in the first 2 years of age, characterized by axial hypotonia, psychomotor regression, ataxia, in addition to several ophthalmological features like, nystagmus, strabismus and a frequent optic atrophy [212, 213]. Twelve North African INAD families with optic atrophy have been reported so far with *PLA2G6* variants, of which 8 were homozygous and 4 compound heterozygous (Additional file 5). A particular mutation p.Val691del, previously reported in other Mediterranean families, was found in 5 Tunisian and one Libyan families, who shared a common haplotype, witnessing a founder effect that occurred 12 generations ago [214].

**Warburg Micro syndrome (OMIM:600,118)** is an autosomal recessive disorder combining neurological symptoms as microcephaly, mental retardation, hypotonia, and eye abnormalities with severe visual impairment with microphthalmia, microcornea, cataract and frequent optic atrophy, in addition to hypogenitalism [215]. More than 32 North African families, mainly from Egypt were reported by several studies [216–219]. Among them, optic atrophy was clearly established in 19 families, with 79% of these families presenting *RAB3GAP1* variants, 2 families with *RAB3GAP2* variants, and 2 others with *RAB18* and *TBC1D20* variants, respectively (Additional file 5).

## Discussion

The data presented in this review provide an extensive overview of IRD and ION phenotypic and genotypic spectrums reported in the literature, regarding 413 North African families. The respective contributions of these 2 groups of diseases show a high predominance of IRD (82.8%) compared to ION (17.2%); fitting with earlier studies from Western countries, suggesting that the origins of blindness are more frequently associated to retinal dysfunctions than to optic nerve degeneration [220].

Nevertheless, by contrast with Europe, North African countries show high rate of consanguinity, as marriage practices are maintained by religion, cultural and socio-economic conditions, in addition to their geographical isolation delimited by the Mediterranean Sea in the North and the Sahara in the South. In this respect, first cousin mating is the most frequent consanguineous marriage in North Africa [13], and progeny of individuals who shared common ancestors are the most likely to be autozygous for recessive variants. This is the case for 71.4% of NS-IRD families for whom investigations of the relationships between parents disclosed a consanguinity, with 82.5% presenting homozygous mutations in recessive genes. For S-IRD, 84.4% were consanguineous, among which 88.2% presented homozygous variants. The same observations were found in ION families, with 84.3% presenting homozygous mutations in nuclear genes.

This high level of consanguinity has further drastic consequences on small endogamous communities, alike the North African Jewish ones, leading to the identification of founder effects associated to variants unusually present at high frequencies in specific genes. For example, although *FAM161A* is not a major cause of RP [39], 18 out of 31 North African Jewish families present a mutation in this gene, among which 14 disclosed with the same homozygous mutation c.1355_1356delCA, 13 living in Morocco. Similarly, 5 out of the 7 Jewish Moroccan RP families presented the same homozygous variant in *EYS*. These peculiarities were also found in non-Jewish North African families, with 4 out of 8 RP families related to the homozygous c.2189 + 1G > T *MERTK* mutation; and virtually all RP families with *CERKL* variants presented the unique c.1133 + 3_1133 + 6delAAGT mutation at a homozygous state, all being of Tunisian ancestry. Similarly, all families reported with RPA were related to the same homozygous 7.36 kb deletion in *RLBP1*. This was also true for all autosomal NS-ION families related to the OPA7 locus, which presented the same *TMEM126A* recessive variant, and for those related to the OPA10 locus, presenting the same *RTN4IP1* recessive variants, both being now encountered respectively in North African and Gipsy families living in Europe, respectively [163, 164, 183–185].

Other biases associated to the clinical spectrum of North African IRD and ION consist in the significant differences in the time course and severity of the visual impairments. Indeed, while S-IRD and S-ION are easily early characterized by severe visual impairment associated to other symptoms, diseases with milder effects on the visual acuity, often occurring later in the life-time, like some NS-IRD and most NS-ION, might be far less clinically diagnosed, as patients can be unaware of their visual difficulties.

This might explain why the proportion of each IRD among the North African population that we report here, is so different compared to what was observed in Western countries. Indeed, RP and STGD diseases, which are the less severe presentations are under-represented, whereas LCA, USH and BBS diseases, which have the worse visual prognosis are significantly over-represented (Table 1).

**Table 1:** Frequencies of IRD subtypes across different countries

Diseases	USA	UK	France	North Africa
	Occurrence and frequencies			
RP	341 (34.1%)	311 (43.1%)	922 (47.1%)	76 (22.2%)
STGD	189 (18.9%)	45 (6.2%)	118 (6%)	8 (2.3%)
USH	81 (8.1%)	37 (5.1%)	207 (10.6%)	61 (17.8%)
CD/CRD	45 (4.5%)	74 (10.2%)	140 (7.2%)	18 (5.3%)
Other macular dystrophies	93 (9.3%)	37 (5.1%)	231 (11.8%)	3 (0.9%)
LCA	14 (1.4%)	18 (2.5%)	52 (2.7%)	41 (12%)
BBS	25 (2.5%)	7 (1%)	23 (1.2%)	46 (13.5%)

USA data are from Stone et al., 2017 [221], based on the study of 1000 families

UK data are from Carss et al., 2017 [30], based on 722 individuals

French data are from Bocquet et al., 2013 [220], based on 1957 individuals

North Africa IRD data are from this review, based on 342 families

Data are provided as the number of families or individuals with the disease and their relative frequency in-between parenthesis within the cohort

This has obvious consequences on the respective frequency of each gene identified in IRD individuals among the North African population, with a lower contribution of variants in the *ABCA4*, *USH2A* and *PRPH2* genes and a higher contribution of variants in the *MYO7A*, *FAM161A*, *GUCY2D* and *MERTK* genes (Table 2).

**Table 2:** Frequencies of IRD genes across different countries

Genes	USA	UK	France	North Africa
	Occurrence and frequencies			
*ABCA4* (AR)	173 (22.8%)	73 (18.1%)	72 (17.3%)	16 (4.7%)
*USH2A* (AR)	76 (10%)	61 (15.1%)	59 (14.1%)	9 (2.6%)
*RPGR* (X-L)	48 (6.3%)	13 (3.2%)	23 (5.5%)	–
*RHO* (AD/AR)	34 (4.5%)	7 (1.7%)	15 (3.6%)	–
*PRPH2* (AD)	32 (4.2%)	6 (1.5%)	20 (4.8%)	1 (0.3%)
*EYS* (AR)	6 (0.8%)	16 (4%)	–	10 (2.9%)
*MYO7A* (AR)	8 (1.1%)	8 (2%)	26 (6.2%)	34 (9.9%)
*FAM161A* (AR)	9 (1.2%)	2 (0.5%)	–	20 (5.8%)
*GUCY2D* (AR)	4 (0.5%)	4 (1%)	1 (0.2%)	19 (5.6%)
*MERTK* (AR)	3 (0.4%)	4 (1%)	1 (0.2%)	10 (2.9%)

USA data are from Stone et al., 2017 [221], based on 760 families

UK data are from Carss et al., 2017 [30], based on 404 individuals

French data are from Bocquet et al., 2013 [220], based on 417 individuals

North Africa data are from this review, based on 342 IRD families

Data are provided as the number of families or individuals and with autosomal dominant (AD) or recessive (AR) variants in the gene considered, and their frequency in-between parenthesis within the cohort with a molecular diagnosis

The same reasons explain why S-ION are proportionally so frequent (88.7%) in this region of the world, while NS-ION are rare (11.3%), and in particular, why no case of isolated Dominant Optic Atrophy has yet been reported in North Africa, although AD ION are at least ten times more frequent than the recessive ones in Western countries [220, 222]. Consequently, these peculiarities explain why the most prevalent ION among the North African families are recessive and syndromic, with the Warburg micro syndrome and the GAPO syndrome predominating in Egypt, and the Infantile neuroaxonal dystrophy 1 predominating in Tunisia, while a single family with dominant S-ION has been reported, with a hereditary motor and sensory neuropathy type-VIA, associated to a heterozygous *ACO2* variant.

Thus, we provided here the first epidemiological data regarding IRD and ION in North Africa, combining clinical and molecular data. Our bibliographical review suggests that the North African populations are far from been fully characterized, because these two groups of diseases are today under-diagnosed, most probably due to the remote location of many patients far away from an Ophthalmologist, the restricted training of Ophthalmologists to rare inherited eye diseases, the difficulties to access local high throughput next generation sequencing, and the limited resources of the population. We also believe that the actual IRD and ION clinical and molecular spectrums in the North African countries are fragmented, as most reports of such information are limited to case reports, or as a part of patient cohorts included in studies from other countries.

Nevertheless, the structural peculiarities of the North African population, with the high rate of consanguinity and the presence of informative large pedigrees with many affected generations, offer outstanding opportunities to identify novel clinical presentations and genes responsible for these inherited eye diseases. With these perspectives in mind, research priorities should now focus on the training of local ophthalmo-geneticists dedicated to the clinical and molecular diagnoses of inherited blinding diseases.

## Conclusion

Inherited retinal dystrophies and optic neuropathies are major causes of early visual impairment that often lead to legal blindness, which are individually rare, but collectively frequent. With a population of some 200 million inhabitants in North Africa, we can expect that some 100.000 individuals (1/2.000 persons) are affected by one of these diseases, a population nowadays far under-diagnosed with respect to the 413 North African families yet reported in medical databases, and compiled here. As both IRD and ION constitute real public health challenges, with major impacts on the educational, autonomy and socioeconomic conditions of affected individuals, future politics should invest in the training of physicians and biologists to improve the diagnosis of inherited ophthalmologic presentations and in equipment dedicated to their molecular diagnosis, to first establish accurate clinical diagnoses, and second constitute well clinically characterized cohorts of individuals eligible to treatments with the emergent therapies for these eye diseases.

## Supplementary Information

**Additional file 1:** Keywords used in the medical Pubmed and Scopus databases.

**Additional file 2:** Phenotypic and mutational spectrum of 194 North African families with non-syndromic inherited retinal dystrophies.

**Additional file 3:** Phenotypic and mutational spectrum of 143 North African families with syndromic inherited retinal dystrophies.

**Additional file 4:** Mutational spectrum of North African families with non-syndromic inherited optic neuropathies.

**Additional file 5:** Phenotypic and mutational spectrum of North African families with syndromic inherited optic neuropathies.

**Additional file 6:** Mutational spectrum of North African families with hereditary mitochondrial diseases with retinal or optic nerve manifestations.

## Abbreviations

IRD: Inherited retinal dystrophy
ION: Inherited optic neuropathy
NS-IRD: Non-syndromic inherited retinal dystrophy
NS-ION: Non-syndromic inherited optic neuropathy
S-IRD: Syndromic inherited retinal dystrophy
S-ION: Syndromic inherited optic neuropathy
AR: Autosomal recessive
AD: Autosomal dominant
RP: Retinitis pigmentosa
RPA: Retinitis punctata albescens
CD: Cone dystrophy
CRD: Cone-rod dystrophy
LCA: Leber congenital amaurosis
EORD: Early onset retinal dystrophy
STGD: Stargardt disease
BMVD: Best vitelliform macular dystrophy
USH: Usher syndrome
USH1: Usher syndrome type 1
USH2: Usher syndrome type 2
USH3: Usher syndrome. Type 3
BBS: Bardet-Biedl syndrome
SLS: Senior-Loken syndrome
JBTS: Joubert syndrome
SJLS: Sjogren-Larsson syndrome
ABL: Abetalipoproteinemia
HS: Heimler syndrome
PBD: Peroxisomal biogenesis disorder
AVED: Familial isolated vitamin E deficiency
FA: Friedreich ataxia
SCA7: Spinocerebellar ataxia type 7
KSS: Kearns-Sayer syndrome
MELAS: Mitochondrial encephalomyopathy, lactic acidosis, and stroke like episodes
MIDD: Maternally inherited deafness and diabetes
LHON: Leber hereditary optic neuropathy
DOA: Dominant optic atrophy
mtDNA: Mitochondrial DNA
WFS: Wolfram syndrome
LS: Leigh syndrome
INAD: Infantile neuroaxonal dystrophy

## Declarations

### Availability of data and materials

All data generated or analyzed during this study are included in this published article and its supplementary files.
