# Supplementary material for: Clinical and genetic spectrums of 413 North African families with inherited retinal dystrophies and optic neuropathies
Source: Orphanet J Rare Dis. 2022 May 12;17:197. doi: 10.1186/s13023-022-02340-7 (PMC9097391; doi:10.1186/s13023-022-02340-7)
Supplement: Supplementary file 1 — Additional file 1: Keywords used in the medical Pubmed and Scopus databases. [file 13023_2022_2340_MOESM1_ESM.docx]

**Inherited retinal dystrophies keywords**

(“Retinal disease” OR “Retinal dystrophy” OR “Retinal degeneration” OR “Retinis pigmentosa” OR “Leber congenital amaurosis” OR “Rod-cone dystrophy” OR “Cone dystrophy” OR “Cone-rod dystrophy” OR “Late-onset retinal degeneration” OR “Bietti crystalline corneoretinal dystrophy” OR “Congenital stationary night blindness” OR “Ogushi disease” OR “Fundus albipunctatus” OR “Achromatopsia” OR “Enhanced S-cone Syndrome” OR “Usher syndrome” OR “Bardet-Biedl Syndrome” OR “Neuronal ceroid lipofuscinosis” OR “Batten disease” OR “Senior-Loken syndrome” OR “Joubert syndrome” OR “Retinitis Pigmentosa with Ataxia” OR “Ataxia with isolated vitamin E deficiency” OR “AVED” OR “Cohen syndrome” OR “Microcephaly Congenital Lymphedema and Chorioretinopathy” OR “Alstrom syndrome” OR “Spinocerebellar Ataxia Type 7” OR “SCA7” OR “Peroxisomal Biogenesis Disorders” OR “Macular degeneration” OR “Macular atrophy” OR “Stargardt disease” OR “Best disease” OR “Pattern dystrophy” OR “Sorsby fundus dystrophy” OR “Malattia Leventinese” OR “Doyne honeycomb degeneration of retina” OR “North Carolina Macular dystrophy” OR “Vitelliform macular dystrophy” OR “Best vitelliform dystrophy” OR “Pseudoxanthoma Elasticum” OR “Choroideremia” OR “Gyrate atrophy” OR “Sveinsson chorioretinal atrophy” OR “Progressive bifocal chorioretinal atrophy” OR “vitreoretinopathies” OR “X-linked juvenile retinoschisis” OR “Wagner syndrome” OR “Snowflake vitreoretinopathy” OR “Retinoschisis” OR “Knobloch Syndrome” OR “Stickler Syndrome” OR “Jalili syndrome” OR “Sjogren-Larsson Syndrome” OR “Refsum Disease” OR “Alport Syndrome” OR “McKusick-Kaufman syndrome” OR “Oculocutaneous Albinism” OR “HARP syndrome” OR “PHARC syndrome”) AND (Morocco OR Moroccan OR Tunisia OR Tunisian OR Algeria OR Algerian OR Libya OR Libyan OR Egypt OR Egyptian OR North Africa OR North African OR Magherb OR Maghrebian) AND (Gene OR Mutation)

**Inherited optic neuropathies keywords**

**1-**

(Optic atrophy OR Optic neuropathy) AND (Morocco OR Moroccan OR Tunisia OR Tunisian OR Algeria OR Algerian OR Libya OR Libyan OR Egypt OR Egyptian OR North Africa OR North African OR Maghreb OR Maghrebian) AND (Gene OR Mutation).

**2-**

(“Leber hereditary optic neuropathy” OR LHON OR “Dominant optic atrophy” OR DOA OR “Kjer’s optic neuropathy” OR “Myoclonic epilepsy, Ragged-Red-Fibers” OR MERRF OR “Mitochondrial encephalomyopathy, Lactic acidosis, and Stroke-Like episodes” OR MELAS OR “Leigh syndrome” OR “Wolfram syndrome” OR “Diabetes insipidus, Diabetes mellitus, Optic atrophy, and hearing Loss” OR DIDMOAD OR “Friedreich ataxia” OR “Spinocerebellar ataxia” OR “Deafness-dystonia-optic atrophy syndrome” OR DDON OR DDS OR “Mohr-Tranebjaerg syndrome” OR “Hereditary spastic paraplegia” OR “SPG7” OR “Hereditary motor and sensory neuropathy” OR HMSNVI OR “HMSN VI” OR HMSN6 OR CMT2A OR “Charcot–Marie–Tooth disease type 2A” OR CMTX5 OR “Behr syndrome” OR “Costeff syndrome” OR “OPA3” OR “3-methylglutaconic aciduria” OR “PEHO syndrome” OR “Mitochondrial DNA depletion syndrome”) AND (Morocco OR Moroccan OR Tunisia OR Tunisian OR Algeria OR Algerian OR Libya OR Libyan OR Egypt OR Egyptian) AND (Gene OR Mutation).
